# A Review of Mathematics Determining Solute Uptake at the Blood–Brain Barrier in Normal and Pathological Conditions

**DOI:** 10.3390/pharmaceutics13050756

**Published:** 2021-05-19

**Authors:** Samuel A. Sprowls, Pushkar Saralkar, Tasneem Arsiwala, Christopher E. Adkins, Kathryn E. Blethen, Vincenzo J. Pizzuti, Neal Shah, Ross Fladeland, Paul R. Lockman

**Affiliations:** 2 Health Sciences Center, West Virginia University, Morgantown, WV 26506, USA; nlshah@mix.wvu.edu; 1 Basic Pharmaceutical Sciences, School of Pharmacy, West Virginia University, Morgantown, WV 26506, USA; sas0040@mix.wvu.edu (S.A.S.); pas0020@mix.wvu.edu (P.S.); taa0025@mix.wvu.edu (T.A.); keblethen@mix.wvu.edu (K.E.B.); vpizzuti@mix.wvu.edu (V.J.P.); rf00015@mix.wvu.edu (R.F.); 3 School of Pharmacy, South University, Savannah, GA 31406, USA; cadkins@southuniversity.edu; 4 Department of Dermatology, School of Medicine, West Virginia University, Morgantown, WV 26506, USA

**Keywords:** pharmacokinetics, blood–brain barrier, brain, magnetic-resonance imaging, preclinical models

## Abstract

The blood–brain barrier (BBB) limits movement of solutes from the lumen of the brain microvascular capillary system into the parenchyma. The unidirectional transfer constant, *K_in_*, is the rate at which transport across the BBB occurs for individual molecules. Single and multiple uptake experiments are available for the determination of *K_in_* for new drug candidates using both intravenous and in situ protocols. Additionally, the single uptake method can be used to determine *K_in_* in heterogeneous pathophysiological conditions such as stroke, brain cancers, and Alzheimer’s disease. In this review, we briefly cover the anatomy and physiology of the BBB, discuss the impact of efflux transporters on solute uptake, and provide an overview of the single-timepoint method for determination of *K_in_* values. Lastly, we compare preclinical *K_in_* experimental results with human parallels.

## 1. The Blood–Brain Barrier

**Figure 1:**
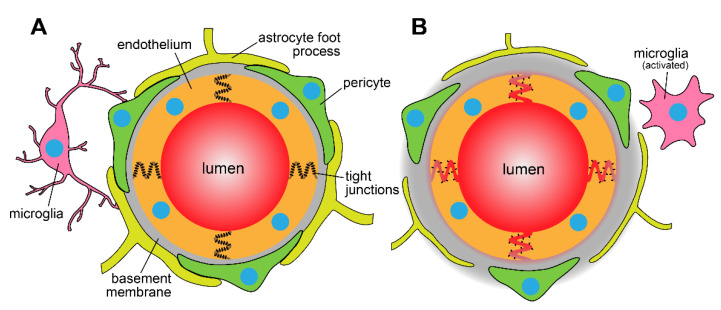
Anatomical differences between (**A**) blood–brain barrier vasculature and (**B**) disrupted BBB. The BBB is characterized by presence of endothelial tight junctions, formed by the tight junction proteins and the adjacent pericytes, microglia and astrocytic foot processes.

The blood–brain barrier (BBB; Figure 1) is the tightly regulated interface of the brain and its microvascular system composed of endothelial cells (ECs), a basement membrane, pericytes, astrocytes, neurons, and microglia. Collectively, these structures cooperate as a highly selective functional barrier capable of regulating the distribution of molecules to brain parenchyma. Claudins, occludins, and junction adhesion molecules (JAMs) form an extensive paracellular barrier between ECs to small molecules, proteins and cells [1]. The ECs at the BBB also exhibit lower rates of transcytosis as a result of non-fenestrated vessels and decreased caveolin-mediated vesicle trafficking compared to the peripheral vascular system [2]. Together, pericytes and ECs secrete an extracellular matrix that surrounds the blood vessels within the brain and forms the basement membrane in which pericytes and astrocytic end-feet become embedded. Astrocytes form the outer layer of basement membrane. The basement membrane serves to facilitate essential intercellular signaling while simultaneously promoting the selective distribution of molecules into the brain parenchyma [3]. The unique characteristics of the BBB impart decreased permeability of solutes in comparison to other vascular networks throughout the periphery.

The primary functions of the BBB are to maintain homeostasis of the brain microenvironment and provide neuroprotection. The BBB preserves the brain microenvironment with influx and efflux transporters. Examples of these include the OCT1, OCT 2, LAT1, and OAT solute carrier protein transporters, which have been suggested to facilitate drug influx at the BBB [4,5],and the P-glycoprotein efflux transporter, which minimizes the passage of many lipophilic solutes from reaching the abluminal membrane of the brain capillary network [6]. Dysregulation of the BBB affects cell signaling, immune cell trafficking, and potential neuronal damage [7]. Selective permeability of the BBB is essential for maintaining central nervous system health, but becomes an obstacle to therapeutic drug distribution into the brain to treat neurological disorders or malignancies of the CNS [8].

## 2. Mapping Drug Kinetics at the Blood–Brain Barrier

Mathematical determination of solute permeability rates across the BBB has not dramatically changed since Patlak et al [9]. described their, at the time, novel model. In their work, the authors proposed a two-compartment model in which influx across the BBB is an irreversible, unidirectional process during the experimental time frame. Model experiments include multiple blood, or plasma, measurements following an intravenous bolus tracer dose across the duration of the experimental time frame from the same subject. Tracer concentration in brain is also obtained at multiple timepoints assumed to be in the linear range of uptake for a given tracer. Plotting the ratio of concentration of tracer in brain (*C_Br_*) at time *t* to concentration of tracer in plasma (*C_pl_*) at time t versus the total exposure of the animal to a given tracer from time *0* to time *t* produces a linear plot as long as the experiment is performed in the range of linear uptake of the given tracer. Regressing these data produces a line with slope of *K_in_*, or the unidirectional transfer constant for the tracer used and y intercept representing the cerebral vascular volume of the test subject measured in units of volume/time/mass, typically as mL/s/g. The expression for movement of solute from the brain capillary network and into the extravascular compartments is given in Equation (1) [9]:

(1) $$C_{Br}=K_{in}\int_{0}^{t}C_{pl}d\tau +\left(V_{0}+V_{pl}\right)C_{pl}$$

where *C_Br_* (tracer/g of brain) is the concentration of tracer in brain, *C_pl_* (tracer/mL) is the concentration of tracer in plasma, *V*_0_ (mL/g) and *V_pl_* (mL/g) constitute the total tracer concentrations within the brain capillary network, and *K_in_* (mL/s/g) is the unidirectional transfer constant for a given solute.

To simplify the kinetic expressions and complement the data from the method above, Takasato and colleagues applied the principles above and created an in situ brain perfusion technique in which the concentration of tracer in the plasma, or in this case the perfusion buffer, remains constant. The in situ brain perfusion technique has several advantages over many traditionally applied barrier integrity protocols. The most striking difference that sets the perfusion technique apart from other methodologies is the ability of the researcher to alter the buffer used to study active transport, protein binding, and a host of other interesting interactions at the BBB. Addition of increasing unlabeled substrate in combination with a constant concentration of radiolabeled substrate can provide insight to the Michaelis–Menten kinetics for a particular substrate–transporter relationship [10]. Additionally, adding serum proteins, adding known inhibitors of efflux transporters [11], or by cooling the perfusion below 37 °C provides the researchers the ability to study the effects of plasma protein binding [12], the affinity of a given substrate for a particular efflux transporter [13], and the impact of temperature [12] on nutrient transport at the BBB. Other notable advantages include avoidance of extracranial metabolism of the solute of interest, less extensive animal surgery, and the possibility to study permeability coefficients over a 10^4^-fold range. The perfusion technique does not replace the intravenous injection technique, but complements the data obtained. The pharmacokinetic expression used to determine tracer uptake in the in situ brain perfusion technique is as follows in Equation (2) [14]:

(2) QBrCpf=KinT+V0

where *Q_Br_* (tracer/g of brain) is the final concentration of solute in the brain, *C_pf_* (tracer/mL) is the tracer concentration is the perfusion buffer, *T* (s) is perfusion time, *V*_0_ (mL/g) is the intercept of the vascular marker used in the experiment (also known as the vascular volume), and *K_in_* (mL/s/g) is the unidirectional transfer constant, obtained from the regressed slope of the brain distribution volume versus time graph.

**Figure 2:**
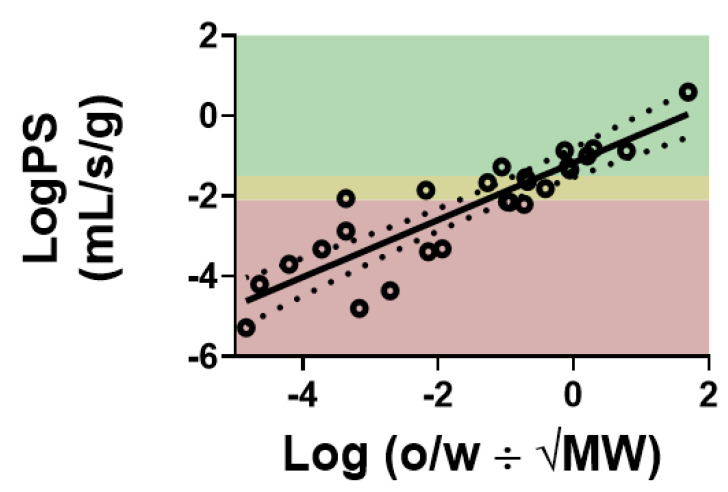
Correlation of solute BBB permeability, indicated by its permeability surface area (PS) product, with the Log(P÷√MW). Compounds with higher lipophilicity have a greater tendency to traverse the BBB. Compounds in the green-shaded area are those with values of 80% of reported cerebral blood flow or high. Compounds in the yellow shaded region indicate those with PS values between 20 and 80% of cerebral blood flow. Compounds with PS values in the red-shaded area are those with reported PS values which are less than 20% of cerebral blood flow. Values compiled from literature reported values of PS [14,15,16,17] *R*^2^ = 0.78.

Data obtained from either of these techniques have been used to make predictive models to determine how fast a novel or understudied solute may permeate across the BBB. A handful of physiochemical properties have been determined to most notably influence BBB permeability: molecular weight, hydrogen bond donors, total polar surface area, and lipophilicity, or LogP. A good agreement exists when plotting the literature, or experimentally verified, permeability coefficients against some variation of the lipophilicity of the same solute. A variety of mathematical alterations to LogP and the use of physiochemical properties in combination with LogP have been used to form predictive estimates of uptake of a multitude of solutes. Figure 2 demonstrates the relative correlation between LogPS and Log (oil/water partition coefficient ÷ √MW). Using the slope from the linear regressed line and the physiochemical properties of a novel substrate, a theoretical or predicted permeability coefficient can be determined.

## 3. Active Efflux at the Blood–Brain Barrier

The BBB dynamically regulates homeostasis and protects the brain from exposure to endogenous entities, toxic drugs and other xenobiotic substances. Multidrug transporters present at the luminal surface of the BBB contribute towards protection by controlling drug distribution and elimination from the brain by ATP-mediated efflux. A majority of these efflux transporters belong to the ATP binding cassette (ABC) superfamily and demonstrate broad affinity for many clinically used drugs based on structure and chemistry of the molecule. Previous studies demonstrate differential expression of several types of ABC transporters at the BBB including P-glycoprotein (P-gp, MDR1, ABCB1), breast cancer resistance protein (BCRP, ABCG2), multidrug resistance protein (MRP1-6, ABCC1-6) and the organic anion transporter (OAT3) [13,18]. Amongst these, the most clinically relevant ABC transporters implicated in prohibiting drug delivery to the brain are P-gp and BCRP. These transporters are responsible for limiting brain access to a wide variety of substrates as a result of extensive expression at the BBB and blood–cerebrospinal fluid barrier (BCSFB) [19,20]. Recent studies demonstrate that these transporters have overlapping affinities for certain substrates which might lead to higher inhibitory effect to drug permeability as opposed to that observed for the individual transporters [21].

The kinetics of efflux can be determined using either of two approaches. Performing in situ brain perfusions as described above to a point of steady state, or to a point where the ratio of tracer in brain to the quantity of tracer in blood does not increase further with time, enables the use of Equation (3) [10].

(3) $$V_{Br}=\frac{K_{in}}{K_{out}}$$

where *V_br_* (mL/g) is the volume of distribution, or the ratio between tracer quantity in brain and blood, *K_in_* (mL/s/g) is the unidirectional transfer constant reflecting the rate at which a substance crosses the brain capillary barrier into the parenchyma, and *K_out_* (s**^−^**^1^) is the rate of efflux of the same solute. A second way to measure the efflux constant is to use a modified in situ brain perfusion in which the brain is preloaded with the solute of interest for a nominal time, and then perfused with tracer-free perfusate for multiple durations. The brain/perfusate ratio can then plotted against time. *K_out_* (s**^−^**^1^) can be determined from these data using the following expression in Equation (4) [10]:

(4) Kout=ln2/t12

where *K_out_* (s**^−^**^1^) is the rate of solute efflux from the brain capillary system, and *t*_1/2_ is the half-life of linear regressed line on the brain/perfusate ratio versus time plot. A similar efflux constant can be determined using either expression so long as the experiments are performed correctly. The in situ brain perfusion technique is a sensitive, effective method that can be used to determine efflux kinetics as described above. Previously, the efflux of thiamine at the BBB was determined using both Equations (3) and (4) [10]. Thiamine efflux did not significantly vary between different brain regions. Interestingly, using predictive models can provide an estimate of *K_in_* as described above. When actual measurements of *K_in_* differ dramatically from predictive models, these compounds are typically subject to efflux. Additionally, in relation to Figure 2, compounds that are effluxed at the BBB typically fall below the linear regressed line indicating that something is preventing them from passing through the BBB as they should based on their physiochemical properties.

Bart et al. used the parameter of distribution volume (DV) to quantify the efflux of P-gp substrate [^11^C]verapamil [22,23]. The efflux of radiolabeled verapamil was measured in rats using PET, and Logan analysis technique was used to calculate the DV. Logan analysis measures the radioactivity of the analyte drug in the region of interest, and the DV is calculated as the slope of the Logan plot. MRI imaging has been used to determine the efflux kinetics after focused ultrasound induced BBB opening. The efflux was found to drop in the FUS-exposed regions, and slowly recovered in a time dependent manner [22].

## 4. Flow- vs. Perfusion-Limited Blood–Brain Barrier Transport

Simple diffusion of compounds across the BBB occurs either paracellularly (between the cells), or transcellularly (through the endothelial cells) [2,24]. Hydrophilic compounds frequently rely on paracellular diffusion due to their poor ability to penetrate the lipid bilayer of the endothelial cell membrane. However, the presence of tight junctions between ECs greatly limits this process. For a compound to cross into the brain transcellularly, it requires an optimal balance between lipophilicity and hydrophilicity to cross the lipid bilayers of the cells as well as the aqueous cytosol. The Lipinski’s ‘Rule of 5′ assists in the prediction of a compound’s BBB permeability. According to this general rule, compounds with fewer than 5 H-bond donors, fewer than 10 H-bond acceptors, a molecular weight less than 500 daltons, and a calculated partition coefficient (logP) value less than 5 are good candidates for BBB permeability [15,25,26]. The logP, determined as the octanol-water partition coefficient of a molecule, denotes its lipophilicity. Generally, a direct relation exists between the passive permeability of a compound across the BBB, and its logP value [27]. This proportionality may not hold true in the case of hydrophilic compounds that undergo transport through specific channels, or lipophilic compounds that are subject to active efflux. Compounds with high lipid solubility can traverse the BBB via simple diffusion process; their entry into the brain is less limited by their physicochemical properties, or carrier-based transport. As a result, the limiting step for the entry of these molecules into the brain is the velocity at which they are supplied to the BBB interface by the blood. Such compounds are said to have a flow-limited BBB permeability. Examples of these compounds include ethanol and diazepam. Of note, these flow-limited compounds are typically used as a measure of cerebral blood flow. Conversely, as a compound’s logP decreases or becomes more negative, its lipid partitioning decreases and, therefore, exhibits a reduction in passive BBB permeability. Their entry into the brain tissue is not dependent on blood flow, and instead depends on their permeability across the BBB, which is indirectly dictated by their physicochemical properties. Such compounds are said to have a permeability limited BBB transport. The transport of solutes occurs over the entire area of the capillary network, and thus to take the surface area into account, the product of permeability and surface area is often used to describe the measure of solute exchange across the BBB, instead of the permeability coefficient alone [27]. Assuming a unidirectional diffusion of solute, the concentration of solute extracted from blood flowing through brain capillaries correlates with the permeability surface area product using the Renkin–Crone equation as follows (Equation (5)):

(5) $$E=1-e^{-PA/F}$$

where *E* is the total solute extraction from blood, *P* is the solute permeability (cm/s), *A* is the total capillary surface area (cm^2^/g of brain), and *F* is the total blood flow (cm^3^/s/g of brain). The above equation can be rearranged solving for the permeability surface area product, PA (Equation (6)):

(6) PA=−Fln(1−E)

**Figure 3:**
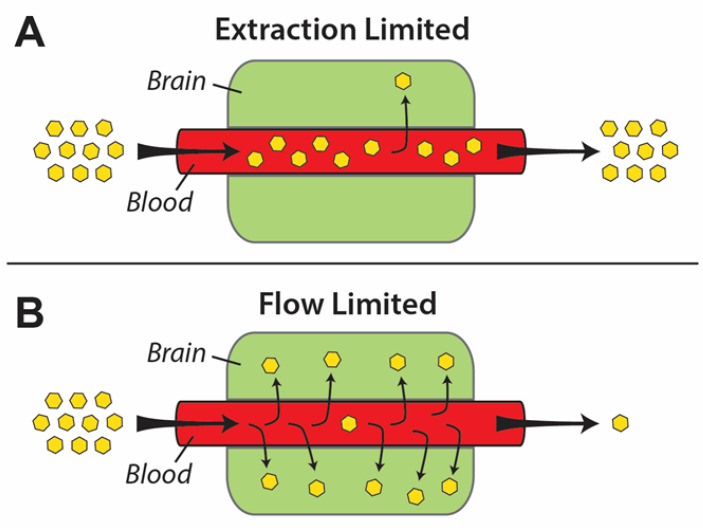
A schematic representation of (**A**) extraction-limited and (**B**) flowlimited solute transfer across the BBB. The physicochemical properties of compounds having extraction-limited permeability are not amenable to BBB transport. Conversely, the transport of highly permeable solutes across the BBB is generally quick, and only limited by how rapidly they are presented to the BBB.

The unidirectional transfer coefficient, *K_in_* (cm/s/g) can be represented as the product of solute extraction and blood flow (Equation (7)).

(7) $$K_{in}=F\times E$$

when the *PA* values are high (*PA*/*F* >> 1), and *K_in_* approaches *F*, solute entry is blood flow limited. When the *PA* values are low (*PA*/*F* << 1), and *K_in_* approaches the permeability surface area product, solute extraction from blood is independent of blood flow and is considered diffusion limited, as depicted in Figure 3 [28,29,30,31]. The values of *PA* can range between 10**^−^**^4^ and 10**^−^**^8^ cm/s. Higher *PA* values of 10**^−^**^5^ to 10**^−^**^4^ cm/s are observed for solutes with a flow-limited transport such as ethanol, caffeine, antipsychotic drugs, and many CNS depressants. Diffusion limited hydrophilic solutes, such as sucrose and mannitol, exhibit PA values several orders of magnitude less, frequently in the ranges of 10^−7^ and 10^−8^ cm/s [32].

## 5. Preclinical Measurements of Blood–Brain Barrier Permeability in Pathological Conditions

Historically, measurements of BBB permeability have been achieved through multiple methodologies. These approaches include the indicator-diffusion, the brain uptake index, the concentration profile analysis, the isolated perfused brain, the intravenous injection, the in situ brain perfusion, and the multiple-time uptake techniques [13,14,31,33,34,35,36,37,38]. Each of these methodologies presents its own limitations ranging from inappropriate assumptions regarding tracer and blood mixing, to inaccurate estimations of poorly or rapidly penetrating solutes, and extensive animal surgery [14]. The in situ brain perfusion is capable of estimating transfer coefficients and evaluating barrier integrity with high fidelity [14,16,28,39,40,41,42]. However, this technique presents limitations regarding its ability to yield reproducible results in disease states with a heterogeneous disruption of the BBB (i.e., brain tumors, stroke, Alzheimer’s disease, etc.). To ascertain these subtle, variable changes in BBB integrity, the single-uptake approach is widely recognized as the preferred methodology [38,43].

The unidirectional transfer constant, *K_in_*, in single-uptake experiments following an intravenous injection of the solute of interest is defined by the relationship in Equation (8) [9,44,45,46]:

(8) $$K_{in}=\frac{C_{br}\left(\tau \right)}{\int_{0}^{t}C_{bl}\;\left(\tau \right)\;dt}$$

where *C_br_* is the concentration of tracer contained in the brain compartment of interest at time *T*, and *C_bl_* is the concentration of solute in blood. The denominator of this expression solves for the area under the curve of the change in plasma concentration from time 0 to time T and indicates total exposure to the solute through the duration of the experiment. The integral of the plasma concentration versus time curve is necessary because the concentration of the test solute in blood changes over time as a result of metabolism and clearance of the tracer. *C_br_* is the total concentration of measurable solute that has left the vascular compartment and distributes to the brain compartment, which is also expressed as the total quantity in brain as follows (Equation (9)):

(9) $$C_{tot}=C_{br}+C_{vas}$$

where *C_tot_* is the total concentration of solute in the brain vascular compartments, and *C_vas_* is the concentration of solute in the vascular space within the brain. Subtraction of the measured *C_vas_* from *C_tot_* provides a reliable estimate of *C_br_*, or the quantity of tracer distribution into brain for a given period of circulation time and unit of tissue mass.

While the pharmacokinetic evaluations in this review provide an estimate of the unidirectional transfer constant for a solute, it has limited insight into the amount of unbound drug in both the blood and brain at a specific time. When considering pharmacokinetics of a solute’s transport from blood to brain it is important to understand that only unbound solute can permeate across the BBB, and the unbound concentration of solute is what drives pharmacodynamic activities [47]. To determine this, an equilibrium micro-dialysis method is used, where a semi-permeable probe is inserted into a specific brain region and perfusate is flowed through an interior probe and allowed to passively diffuse across the outer semipermeable membrane. The dialysate is then measured by collection from the outlet tube [48,49]. Briefly the equilibrium constant K,p,uu (unbound partition coefficient) is determined as follows in Equation (10):

(10) $$K_{p,uu}=\frac{AUC_{u,brain}ISF}{AUC_{u,plasma}}$$

where *AUC_u,brain_* and *AUC_u,plasma_* represents the total exposure of unbound drug in brain and plasma, respectively [49,50]. Determining *K_p,uu_* provides information on the concentration of drug freely able to act within the brain parenchyma. This measure accounts for tissue binding affinity and the properties of active and passive transport across the BBB [51], though it does not directly measure BBB transport constants. Values of *K_p,uu_* are reported to range from as low as 0.02 and 3. Contextually, solutes with high BBB permeability/equilibrium such as diazepam and oxycodone have a *K_p,uu_* value of 1 and 3, respectively [52,53]. Conversely, baclofen and morphine, solutes with poor BBB penetration and equilibrium, have a reported *K_p,uu_* values of 0.02 and 0.29 respetively [54,55].

## 6. Clinical BBB PK in Disease States and Preclinical Model Translatability

Measurement of BBB permeability and disruption in humans is not as direct as preclinical models but is readily achieved with advanced imaging techniques such as dynamic contrast-enhanced MRI (DCE-MRI), often employed in oncology and stroke imaging studies [56,57,58,59]. This type of imaging provides researchers and clinicians with estimates of *K_trans_* to quantify BBB permeability. As defined by Tofts, *K_trans_*, with units of min**^−^**^1^ (or time**^−^**^1^), is a volume transfer constant between blood plasma and extravascular, extracellular space, predominantly intended for use with tracers that do not readily enter intracellular compartments (i.e., non-lipophilic tracers) [60]. Measures of K_trans_ in DCE-MRI studies are often calculated utilizing the extended Tofts-Kety (ETK) model, but can also be estimated from linearized Patlak plots of concentration versus time data; however, this method assumes negligible backflow of contrast agent from extravascular spaces into blood vessels during the scanning period [60,61,62,63]. Unlike *K_in_* values, *K_trans_* is expressed in units of time**^−^**^1^ because each concentration term is based solely on volumetric signal and cannot be normalized to brain tissue mass, as is the case for preclinical determinations of *K_in_*. The ETK and linearized Patlak model equations frequently used in this setting are displayed below (Equations (11) and (12)) [60,61,64]:

(11) $$C_{br}\left(t\right)=K_{trans}\int_{0}^{t}C_{br}\left(\tau \right)e^{-k_{el}\left(t-\tau \right)}d\tau +f_{vasc}C_{bl}\left(t\right)\;(\mathrm{ETK}\;\mathrm{Model})$$

(12) $$C_{br}\left(t\right)=K_{trans}\int_{0}^{t}C_{br}\left(\tau \right)d\tau +f_{vasc}C_{bl}\left(t\right)\;(\mathrm{Linearized}\;\mathrm{Patlak}\;\mathrm{Model})$$

where *C_br_* is the concentration of contrast agent in the brain compartment, *C_bl_* is concentration in the blood, *f_vasc_* is the volume fraction of vasculature in the tissue, and *k_el_* is the elimination rate constant from brain to blood compartments. In the linearized Patlak model, the elimination rate constant is ignored as discussed previously. After pre- and post-contrast infusion scans are obtained, non-linear (ETK) and linear (Patlak) least squares regression of these parametric equations are used to estimate *K_trans_*. The estimates of permeability changes or barrier disruption provide important clinical implications regarding many disease states, including cancer and stroke.

Brain tumors, whether primary or metastatic, heterogeneously disrupt local brain microvascular architecture and function which results in variable increases to passive permeability of the blood–tumor barrier (BTB) [65,66]. Notably, measures of *K_trans_* in human gliomas are often elevated by orders of magnitude compared to healthy contralateral brain tissue and has been shown to correlate with glioma grade [61,67]. Trends in permeability increases, indicated by fold-changes in *K_in_* values for lesioned versus normal brain are also observed in mouse models of glioma and breast cancer brain metastasis [38,68,69]. Agreement in trends between the estimates of fold-enhancement indicates that these mouse models faithfully capture the important underlying factors that dictate BBB permeability changes observed in humans.

In the case of acute ischemic stroke, clinical studies employing DCE-MRI have found significant increases in the value of *K_trans_* in affected regions compared to normal contralateral areas of brain parenchyma [70]. Elevated fold changes in *K_trans_* between affected and unaffected regions (in one study, approximately 3.5 for early post-stroke and approximately 23 for 5–7 day follow-up) [70] are similar in magnitude to the changes in relative permeability of the BBB to dye observed in a rat model of ischemic stroke (approximately 15 fold change) [71]. This indicates that BBB permeability measurements in rodent stroke models effectively mimic the types of changes in permeability between stroke-affected and unaffected brain regions in human.

Previously discussed disease states provide validation and justification for the continued use of mouse and rat models due to their observed pathophysiological mimicry to clinically observed BBB function in these disease settings. While absolute values observed in these preclinical models do not scale directly to clinical values, the observed fold-changes in BBB permeability appear to translate consistently. Preclinical experiments studying BBB permeability across various diseases should be designed in light of the importance of appropriate normal parenchymal controls, as such measures set the baseline for determination of clinically translatable and meaningful fold-change measurements.

**Figure 4:**
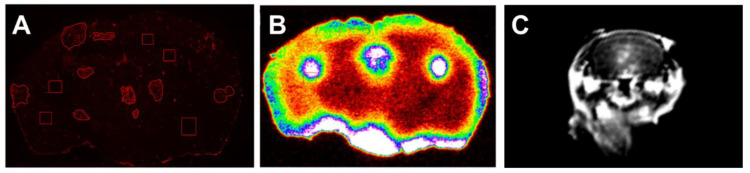
Differential tracer uptake in various imaging modalities. Accumulation of Texas Red 3K (**A**) and 14C-aminoisobutyric acid (**B**) in brain metastases of breast cancer using fluorescent and phosphorescent quantitative imaging. (**C**)T1 cortical Turbo Spin Echo MRI indicating gadavist enhancement in lesions within the brain.

Interestingly, the magnitude of fold-change is notably different among preclinical and clinical determinations of passive permeability. One proposed source of variance that may be distinct other than the mathematics applied to each model is the tracer used in each study. Tracer or particle charge, size, polar surface area, among other properties, all variably affect BBB transport. Keeping in mind these parameters, a difference in fold-change of 9.2 for TxRed and 47.7 for Gd-DPTA both in a glioma model may not be all that different given the difference in physiology of mice and humans, as well as the immune system status in various animal models. Both provide measures of barrier damage, but also are indicative of the size, charge, and other chemical properties of that molecule. Figure 4 shows the difference in uptake of three distinct solutes detected through three separate imaging modalities. The BBB is consistent from species to species, at least in the case of humans and small rodents regarding cellular makeup and the rate of uptake of solutes at the BBB. However, what does change among specifies is the specific transporter composition (i.e., BCRP, P-gp, MRP1, etc.) at the BBB. While these data may indicate differences among methods, other correlates of animal and human data can be provided regarding therapeutic efficacy and brain tumors.

## 7. Conclusions

The BBB dictates the kinetics of solute transfer into and out of the brain, having implications over the extent of drug distribution and treatment efficacy. This review outlines the techniques and the mathematical models commonly used to determine solute influx and efflux across the BBB. These techniques, such as in situ perfusion and Patlak modeling have found application in preclinical as well as clinical research. Determination of the rate of drug transfer across the BBB bears great significance during the preclinical and early stage CNS drug development process. Application of such methods could help predict drug disposition, allowing for optimal treatment of CNS pathologies. Furthermore, this review is limited in its capacity, largely describing the unidirectional transfer rate at which a particular solute crosses the BBB. Not described herein are other sophisticated methods that also aim to determine BBB transport such as the use of microdialysis and serial CSF sampling. A complex multimodal approach using a variety of uptake methodology would be suitable for a more complete understanding of BBB transport for any given solute.

## Data Availability

All figures and data can be obtained by contacting the corresponding author, Paul R. Lockman.
